# Fragrance-Free Policies in Practice: Identifying Perceived Implementation Gaps Through Lived Experience in a Qualitative-Dominant Mixed-Methods Study

**DOI:** 10.3390/ijerph23081077

**Published:** 2026-08-19

**Authors:** Sudipta Roy, Nene A. Diallo, John Molot, Robert Lattanzio, Riina Bray, Jennifer Armstrong, Rohini Peris

**Affiliations:** 1 Association pour la santé environnementale du Québec-Environmental Health Association of Québec (ASEQ-EHAQ), Saint-Sauveur, QC J0R 1R1, Canada; sudipta@aseq-ehaq.ca (S.R.); ndiallo@aseq-ehaq.ca (N.A.D.); 2 Faculty of Medicine, University of Ottawa, Ottawa, ON K1G 5Z3, Canada; jmolot@rogers.com; 3 ARCH Disability Law Centre, Toronto, ON M5J 2H7, Canada; robert.lattanzio@arch.clcj.ca; 4 Environmental Health Clinic, Women’s College Hospital, Toronto, ON M5S 1B2, Canada; riina.bray@rogers.com; 5 Department of Family and Community Medicine, University of Toronto, Toronto, ON M5G 1V7, Canada; 6 Ottawa Environmental Health Clinic, Ottawa, ON K2H 5A8, Canada; dr@oehc.ca

**Keywords:** indoor air quality, multiple chemical sensitivity, fragrance-free policy, accessibility, built environment

## Abstract

**Highlights:**

**Public health relevance—How does this work relate to a public health issue?**

- Indoor air quality and fragrance-related exposure affect health, accessibility, and participation in daily activities.
- This study identified gaps and inconsistencies of existing fragrance-free policies in practice.

**Public health significance—Why is this work of significance to public health?**

- Gaps in policy clarity, accountability, and implementation may limit the effectiveness of fragrance-free policies.
- The findings contribute evidence to inform future fragrance-free policy development and implementation efforts.

**Public health implications—What are the key implications or messages for practitioners, policy makers and/or researchers in public health?**

- Comprehensive fragrance-free policies may improve accessibility and reduce barriers to participation in public and institutional settings.
- Stronger fragrance-free policies may support healthier indoor environments, improve access to healthcare, and promote better health outcomes.

**Abstract:**

Background: Indoor air quality (IAQ) represents a key public health and accessibility concern. Improving IAQ through policies and practices that reduce exposure to fragranced products and other indoor air contaminants may enhance accessibility and support equitable participation for individuals with Multiple Chemical Sensitivity (MCS). Methods: Using a qualitative-dominant mixed-methods design, we examined IAQ accessibility barriers and explored perceived implementation and accessibility gaps in existing fragrance-free policies among sixty individuals with MCS and related IAQ exposures across ten focus groups. Qualitative and quantitative data were collected in parallel, with thematic analysis conducted in NVivo. A structured IAQ and policy survey was administered to quantify reported experiences and policy impacts. Results: Approximately 63.3% of focus group participants reported dissatisfaction with the indoor environments they encountered. Qualitative findings highlighted indoor environments as inaccessible, inadequately controlled, and associated with ongoing exposure to fragranced products as well as other indoor pollutants. These findings indicate that current approaches often fail to ensure accessibility, leaving individuals with MCS exposed and excluded from full participation in workplaces, healthcare settings, and other public spaces. Conclusions: Comprehensive fragrance-free policies incorporating source control, education, ventilation, and accountability mechanisms may reduce exposure while improving accessibility and inclusion across the built environment.

## 1. Introduction

Indoor air quality (IAQ) is a key determinant of public health and accessibility within the built environment. Among the populations most affected by poor IAQ are individuals with multiple chemical sensitivity (MCS) and related conditions, who experience adverse health effects from low-level exposure to common airborne chemicals, including those emitted from fragranced consumer products [1,2]. These exposures can trigger respiratory, neurological, and systemic symptoms, significantly limiting individuals’ ability to access workplaces, healthcare settings, and public spaces [1,2,3,4]. According to preliminary 2025 Canadian Community Health Survey (CCHS) data, over 3.1 million (9.4%) Canadian adults reported having MCS, while approximately 900,100 (2.7%) reported receiving a diagnosis [5]. As such, MCS is increasingly recognized not only as a health condition but as a disability arising from the interaction between individuals and environmental conditions within the built environment [2,6,7].

In response to the growing concerns regarding IAQ and accessibility, fragrance-free policies have been widely adopted across institutions, including hospitals, universities, government buildings, and workplaces [8]. While fragrance-free policies have become increasingly common, relatively little research has examined how these policies function in practice from the perspective of those they are intended to protect. Policy presence alone does not guarantee effective implementation, and formal fragrance-free designations may not reflect actual environmental conditions experienced by building occupants. Understanding lived experience is therefore essential for understanding how existing fragrance-free policies are experienced in practice and how participants perceive their influence on accessibility and meaningful participation in everyday settings.

Drawing on focus group findings, survey findings, and existing IAQ evidence as contextual support, this study examined the barriers individuals with MCS and other disabilities affected by IAQ encounter when accessing the built environment; assessed the impacts of these barriers on health, access to services, and social participation; and identified recommendations for improving policies and practices to better support accessibility and inclusion.

## 2. Materials and Methods

### 2.1. Study Design

We used a qualitative-dominant mixed-methods design to examine accessibility barriers associated with IAQ and to explore perceived implementation and accessibility gaps related to fragrance-free policies in practice. Qualitative focus groups and a quantitative building occupant survey were conducted in parallel as complementary components of a broader IAQ research study examining fragrance exposure, policy implementation, and accessibility in the built environment. The focus groups explored the lived experiences of individuals with MCS and related IAQ exposures, while the quantitative survey assessed fragrance-free policy awareness, implementation, and perceptions of indoor environments among building occupants. The two datasets were collected from distinct populations, analyzed independently, and integrated during interpretation. Focus groups served as the primary source of data, while the building occupant survey was included to characterize the organizational and environmental context in which fragrance-free policies were implemented, providing complementary context for interpreting the accessibility barriers described by the individuals with MCS.

### 2.2. Study Participants

A total of 60 participants took part in the qualitative components across ten focus groups, representing a diverse range of ages, socioeconomic backgrounds, geographic regions, and health experiences. Participants were included in the study if they met the following inclusion criteria: (i) age ≥ 18 years, (ii) reside in Canada, (iii) experience symptoms on exposure to fragranced products or indoor air contaminants, (iv) can communicate in English or French, and (v) consented to being contacted to participate in the focus group. Recruitment was conducted online through social media, organizational networks, and project partners. Participants included individuals with MCS as well as those reporting symptoms associated with fragrance and IAQ exposures.

Survey respondents were general building occupants, distinct from the focus-group participants recruited for the qualitative component of this study. A subset (7 of 48 respondents, 14.6%) self-identified as having a diagnosed or self-diagnosed MCS; the remainder represented the broader occupant population of the same office buildings where IAQ field assessments were conducted as part of the broader research study. Occupant survey responses were collected between January and July 2024, parallel to the broader IAQ field assessment period (December 2023–June 2024).

### 2.3. Data Collection

Eligible participants were provided with study information and consent materials prior to data collection. With informed written consent, participants were invited to pre-scheduled virtual sessions via Zoom and were instructed to keep their cameras off to maintain anonymity. Data were collected through semi-structured focus groups using a standardized interview guide. Respondents were encouraged to describe their lived experiences with indoor environments, including specific triggers, barriers encountered in different settings (e.g., workplaces, healthcare, public spaces), and perceived gaps in existing policies or accommodations. The duration of each focus group was approximately two hours. A trained moderator facilitated each session, with a second researcher present to support documentation. All sessions were audio-recorded and transcribed verbatim. Transcripts were subsequently reviewed and cleaned to remove identifying information prior to analysis. Data collection and analysis occurred concurrently. Saturation was considered achieved when successive focus groups yielded no substantially new themes, concepts, or explanatory insights relevant to the study objective.

In addition to the focus groups, a structured IAQ and policy survey was administered anonymously to building occupants during the IAQ field assessments conducted in office buildings across seven Canadian provinces (Ontario, Quebec, British Columbia, New Brunswick, Manitoba, Nova Scotia, and Saskatchewan). The survey documented experiences related to indoor environments, fragrance-free policies, and accessibility. Survey findings were analyzed descriptively and used as contextual evidence to complement and support interpretation of the qualitative findings. Focus groups and the survey represent separate components of the broader research study. Because the survey was distributed using an anonymous online link through building contacts rather than a tracked list of individual occupants, an exact response rate relative to the number of occupants invited could not be calculated. However, to prevent duplicate responses from single occupants, the survey was configured to accept one submission per device/browser, using browser cookies.

### 2.4. Data Analysis

#### 2.4.1. Qualitative Analysis

Focus group transcripts were analyzed using thematic analysis, combining inductive and deductive approaches. An initial coding framework was developed and iteratively refined through multiple readings. Two researchers independently coded the transcripts. Coding differences were resolved through internal discussions and consensus building, resulting in refinement of the coding framework and re-coding where necessary. Coding was conducted using NVivo 14 software, and coder triangulation was applied to enhance analytic rigor and consistency. Themes were organized into key domains, reflecting both environmental and social determinants of accessibility.

#### 2.4.2. Quantitative Analysis

Survey data from the IAQ and policy questionnaire completed anonymously by building occupants during the IAQ assessments were analyzed descriptively to characterize perceptions of indoor environments, fragrance-free policies, and accessibility. Descriptive statistics were used to summarize respondent characteristics and reported experiences. Categorical variables are presented as frequencies and percentages. Exploratory Fisher’s exact tests were performed in RStudio (Version 2026.07.1+147) to compare the distribution of participant sociodemographic characteristics, baseline chronic health conditions, and self-reported health symptoms between respondents from buildings with fragrance-free and non-fragrance-free policies. Because these analyses were exploratory and unadjusted, they were intended to identify potential differences between groups rather than evaluate the effectiveness of fragrance-free policies. The findings were used to complement and contextualize the qualitative findings within the mixed-methods design.

### 2.5. Data Validation

We used several strategies to enhance the credibility of the findings. Methodological triangulation was achieved through the integration of evidence from the IAQ and policy survey and focus group findings. Consensus among coders was achieved through collaborative coding by multiple researchers and iterative refinement of themes through comparison and discussion. Participant feedback was used to confirm that the findings accurately reflected lived experiences and to enhance the credibility of the analysis.

### 2.6. Epistemological Framework

The study was informed by a critical realist and social constructionist perspective, recognizing that some physical barriers (e.g., the presence of fragrances, VOCs, poor ventilation) objectively exist in the environment and have real health impacts, while the barriers and emotional impacts affecting these individuals are socially constructed or are a result of human social dynamics. This framework supports conceptualizing MCS as both a health condition and an accessibility issue arising from interactions between individuals and their environments.

### 2.7. Ethical Considerations

This study was approved by the Women’s College Hospital Research Ethics Board (REB Protocol No. 2023-0030-E). Informed consent was obtained from all participants prior to data collection. Participation was voluntary, and participants could withdraw from the study at any time without consequence until data had been de-identified and incorporated in the analysis. Given the sensitive nature of participants’ health experiences, efforts were made to ensure a respectful and supportive environment during discussions. Focus groups were conducted anonymously using participant identifiers rather than personal names, and all data were de-identified during transcription and analysis to protect confidentiality. Audio recordings, transcripts, and study data were stored securely and accessible only to authorized members of the research team.

## 3. Results

### 3.1. Focus Group Sample Characteristics

A total of 60 participants took part in the study across ten focus groups (Table S1). Participants were recruited from across Canada, with representation from multiple provinces, most notably Ontario and Quebec (61%), as well as smaller numbers from other regions. Approximately 78% of participants were English-speaking, and 63% were White–North American by race/ethnicity. However, all were established Canadian residents. The sample was predominantly female (90%), with the majority of participants aged between 36 and 64 years (70%). Indicators of economic vulnerability were common within the study population. Half of the participants reported annual incomes below $50,000 CAD, including 33% reporting incomes below $25,000 CAD per year. In addition, 62% of participants reported being unemployed. Participants also reported a high burden of comorbid conditions, which most commonly included headache/migraine (*n* = 28, 46.7%), followed by MCS (*n* = 22, 36.7%), chronic fatigue (*n* = 19, 31.7%), asthma (*n* = 14, 23.3%), fibromyalgia (*n* = 13, 21.7%), anxiety (*n* = 10, 16.7%), dermatitis (*n* = 10, 16.7%), eczema (*n* = 7, 11.7%), and cardiovascular complications (*n* = 7, 11.7%). Neurological and cognitive symptoms—including brain fog, dizziness, trouble concentrating, and seizures—were also present across the sample.

Overall satisfaction with the indoor environments was low among participants. More than half of the focus group participants (*n* = 38, 63.3%) reported being dissatisfied or extremely dissatisfied with the indoor environments they encountered, which supports the qualitative data indicating that many indoor environments were experienced as inaccessible, inadequately controlled, and associated with ongoing exposure to fragranced products and other indoor pollutants.

### 3.2. Building Occupants Survey Descriptives

The anonymous survey was completed by 48 building occupants representing 17 office buildings across seven Canadian provinces. Thirty-two respondents (66.7%) worked in buildings with a fragrance-free policy, whereas 16 (33.3%) were employed in buildings without such a policy. Detailed descriptive statistics for respondent characteristics, fragrance-free policy awareness and implementation, indoor environmental perceptions, and self-reported health outcomes are presented in Supplementary Materials (Tables S2–S5). Exploratory analyses showed no statistically significant differences between the groups for any sociodemographic characteristic, chronic health condition, or self-reported health symptoms assessed (all *p* > 0.05).

### 3.3. Themes in Barriers: Systemic Failure Pathways in Policy Implementation

The World Health Organization (WHO) defines barriers as factors in a person’s environment that limit functioning and create disability through their absence or presence, which include inaccessible physical environments, a lack of appropriate assistive technology, negative attitudes of people towards disability, services, systems and policies that are either non-existent or that hinder the involvement of all people with a health condition in all areas of life [9]. Participant accounts suggested that the barriers they experienced were not isolated problems but reflected interconnected weaknesses in policy design and implementation that collectively contributed to persistent exposure and reduced accessibility.

#### 3.3.1. Attitudinal Barriers

Attitudinal barriers, as recognized in accessibility legislation [10], encompass negative assumptions, skepticism, and institutionalized misunderstandings about disability that shape decision-making and restrict equitable participation. Analysis of participant narratives revealed that attitudinal barriers were the most prevalent category of reported experience discussed by 43 of the 60 participants. One participant explained, “I think it’s the attitudinal barriers that are the worst”, emphasizing that social invalidation itself became a significant barrier. Within this theme, attitudinal barriers may manifest through the following interconnected sub-themes:

- Sub-theme 1: Lack of awareness

Within attitudinal barriers, lack of awareness emerged as the most consistently reported sub-theme. In the focus groups, a pervasive lack of awareness of the impacts of everyday products on one’s health was consistently highlighted, particularly in workplaces, healthcare settings, and shared public environments, where fragranced products were often treated as harmless or as a matter of personal preference rather than as potential accessibility barriers. Although policies frequently existed, organizations failed to provide adequate education on MCS and fragrance exposures. One participant noted, “They all think that everything’s regulated by Health Canada. Therefore, because it’s available, it must be safe”. This lack of education may have contributed to inconsistent product use and poor environmental practices.

- Sub-theme 2: Skepticism and denial

Limited awareness was frequently associated with skepticism and denial regarding the legitimacy of MCS, with participants reporting that their symptoms were questioned, minimized, or dismissed by employers, healthcare providers, co-workers, and service staff. Because many symptoms were invisible and exposure thresholds varied, those affected were often perceived as exaggerating or overreacting. One participant stated, “They got it in their head that I just wanted to work from home”. This skepticism may have weakened organizational willingness to implement meaningful accommodations. When leadership or staff doubted the legitimacy of exposures, enforcement became inconsistent and responsibility shifted onto affected individuals to manage their own risk.

- Sub-theme 3: Stigmatization

Focus group narratives suggested that skepticism may have contributed to broader stigmatization, where affected individuals felt socially labeled or marginalized based on their condition or accommodation needs. People may be perceived as overly sensitive, difficult, demanding, or disruptive when requesting fragrance-free or environmentally safe spaces. This stigmatization produced important downstream effects resulting in social isolation, emotional distress, reluctance to disclose health conditions, and reduced participation in workplaces, healthcare settings, educational institutions, and public environments. Many individuals intentionally avoided healthcare appointments, workplaces, meetings, or public spaces because repeated negative interactions made disclosure emotionally and socially exhausting. In many cases, these experiences were linked to institutional cultures that prioritized personal preference and convenience over accessibility. Fragrance use remained socially normalized, while accommodation requests were treated as exceptional burdens. Consequently, policies lacked the cultural support necessary for consistent implementation.

- Sub-theme 4: Discrimination

Participants shared experiences of discrimination, including refusal to modify environments, exclusion from meetings or public spaces, barriers to employment, or inadequate access to healthcare and services. They repeatedly emphasized that policies failed not because they were absent, but because enforcement mechanisms were weak, inconsistent, or entirely lacking. Many described scenarios in which fragrance-free signs were posted while staff continued to wear scented products or fragranced cleaning agents remained in routine use. Frustration and distrust were reported by participants when organizations publicly promoted accessibility measures that were not reflected in environmental conditions. Although environmental measurements were not collected as part of the present study, the IAQ findings provide contextual evidence that incomplete implementation of fragrance-free policies may result in continued chemical exposures, consistent with participants’ reports of ineffective policy enforcement.

- Sub-theme 5: Conflict

In many cases, requests for accommodation generated interpersonal conflict, particularly when fragrance-free practices were viewed as personal preferences rather than accessibility measures. These tensions often reinforced participants’ feelings of social isolation and emotional distress, particularly when organizations failed to implement clear accessibility policies, education, and environmental practices that supported equitable participation.

- Sub-theme 6: Unwillingness to Accommodate

Participants’ narratives suggested that unwillingness to accommodate was associated with the cumulative outcome of earlier systemic failures. Unclear definitions, inadequate education, skepticism, stigma, and weak enforcement collectively shaped environments in which accommodations were treated as negotiable rather than necessary. Even in environments with fragrance-free guidelines, accommodation was often inconsistently applied or ignored altogether. As one participant explained, the problem was not simply the existence of fragranced products, but the “attitudinal poisoned environment” surrounding them. The resulting unwillingness to accommodate reinforced the perception that accessibility policies were symbolic rather than operational.

#### 3.3.2. Physical Barriers

Physical barriers refer to environmental and structural conditions that restrict participation through exposure pathways, spatial design, or building systems that fail to maintain accessible indoor air conditions [11]. Individuals identified cleaning products (*n* = 50), perfumes and scents (*n* = 47), laundry products (*n* = 41), and paints (*n* = 33) as the most persistent sources of exposure across workplaces, healthcare settings, homes, and public spaces. Additionally, mold or water-damaged buildings (*n* = 31), construction or renovation materials (*n* = 30), and furniture or carpeting (*n* = 25) were identified as contributing to accessibility challenges.

- Sub-theme 1: Indoor environment characteristics

Indoor environment characteristics were consistently identified as major contributors to symptom exacerbation. Participants described physical features of indoor environments including building materials and furnishings, poor ventilation, high humidity, enclosed spaces, recirculated air systems, and inadequate air filtration as contributing to accumulation of pollutants. IAQ evidence aligned with these experiences, indicating that indoor environments may continue to contain elevated levels of VOCs and other pollutants when fragranced or other VOC-emitting products remain in use. Importantly, these exposures were reported even in environments where fragrance-related policies formally existed, suggesting that environmental standards were often insufficiently implemented or monitored.

- Sub-theme 2: Built environment

Our study recognized broader failures within built environments, particularly in workplaces, healthcare facilities, educational institutions, public buildings, shared housing, and transportation systems. Many environments were perceived as inaccessible because exposure prevention was not integrated into building management or accessibility planning, highlighting how physical and social dimensions of exposure interacted within shared indoor spaces. IAQ survey findings further demonstrated that shopping malls or retail stores (*n* = 37), places of worship (*n* = 35), entertainment venues (*n* = 35), and hotels or other accommodations (*n* = 34) were among the most commonly identified inaccessible environments, followed by hospitals and medical facilities (*n* = 31), public transport (*n* = 30), and workplaces (*n* = 29). These findings suggest that exposure risks extend across a wide range of everyday environments, limiting participants’ ability to safely access essential services, employment, healthcare, education, transportation, and social participation.

- Sub-theme 3: Products and materials

A major source of ongoing exposure involved institutional procurement practices and personal product choices, particularly fragrances and perfumes, laundry products (including detergents and fabric softeners), and cleaning products. Those products and materials were repeatedly reported as persistent triggers in nominally fragrance-free environments. These findings pointed to failures in procurement controls, with organizations lacking clear standards governing which products are permitted within indoor spaces.

#### 3.3.3. Systemic Barriers

Systemic barriers encompass institutional policies, administrative procedures, and governance practices that indirectly or directly restrict equitable participation [11].

- Sub-theme 1: Availability of healthy alternatives

In this study, limited availability of healthy alternatives was observed as a major structural barrier. Although fragrance-free or lower-emission products existed, these products were often inconsistently available to the general public. As one participant summarized, “there are options… it’s just, how do you get them?” Also, there were challenges accessing healthier building materials, safer heating systems, and low-emission housing options. Participants described the lack of alternatives as limiting their ability to avoid harmful products, suggesting that these experiences reflected broader systemic gaps in accessibility to safer environmental alternatives that extended beyond individual accommodations.

- Sub-theme 2: Healthcare accessibility

Barriers within healthcare accessibility were especially prominent. Respondents described healthcare settings as one of the clearest examples of systemic policy failure, where settings intended to support health instead became sources of exposure and exclusion. Despite the heightened vulnerability of individuals with MCS and related conditions, hospitals, clinics, physician offices, and home care environments frequently lacked effective fragrance-control measures, accommodations, or staff awareness regarding IAQ-related disabilities—health conditions such as MCS, asthma, migraines, and other chronic conditions that are triggered or worsened by indoor air contaminants including VOCs and fragranced products. Many reported delaying or avoiding medical care because healthcare environments themselves triggered symptoms. One participant explained that “even regular doctors, they don’t understand this because a lot of the symptoms mimic other chronic illnesses.” These experiences highlight major gaps in clinical recognition and treatment.

- Sub-theme 3: Lack of policy enforcement

A central theme across participant accounts was the lack of policy enforcement. Although fragrance-free policies, signs, educational campaigns, and workplace communications were often formally in place, these measures were largely symbolic because organizations failed to monitor compliance or apply consequences for repeated violations. One participant explained that “We have an extremely strict scent-free policy, but it’s just not enforced.” This enforcement gap allowed earlier failures in education, communication, and procurement to persist unchecked, resulting in continued exposure despite the presence of a policy.

- Sub-theme 4: Lack of industry accountability

A recurring concern was the broader lack of industry accountability, particularly regarding product formulation, chemical disclosure, and fragrance marketing practices. Manufacturers were perceived as insufficiently regulated and able to continue marketing fragranced products without transparent disclosure of potential health impacts or emissions. This may have contributed to widespread uncertainty regarding product safety and reinforced the normalization of indoor chemical exposures.

- Sub-theme 5: Legal and accommodation gaps

Interviewees described persistent legal and accommodation gaps limiting their ability to secure meaningful protections in workplaces, housing, education, and healthcare systems. Although some organizations attempted accommodations, there was inconsistent recognition of MCS-related disabilities and limited enforcement of accessibility protections. One participant described being moved between workplaces after becoming ill in a newly constructed “sick building”, with similar VOC exposures from new carpets, furniture, and building materials, ultimately resulting in medical leave. Another participant stated that although their employer “tried to accommodate” them, the accommodation itself remained incompatible with their health needs due to persistent environmental exposures. Others noted resistance from co-workers or institutions who viewed fragrance use as an individual right that outweighed accommodation needs. Together, these narratives suggested that policy failures extended beyond individual environments and reflected broader institutional and regulatory deficiencies that perpetuated exclusion and ongoing exposure.

#### 3.3.4. Informational Barriers 

Informational barriers arise when environmental risks, ingredient disclosures, or policy expectations are unclear, inaccessible, or inconsistently communicated [10]. In other words, informational barriers occur when people with disabilities are unable to access or receive the information they need (due to formats or communication methods that are inaccessible or not tailored to their needs). Participants described persistent uncertainty regarding which products, environments, or behaviors were actually safe under existing policies.

- Sub-theme 1: Product label transparency

One of the most frequently discussed issues involved limited product label transparency. Significant difficulties were reported in identifying products that would not trigger symptoms because ingredient lists were incomplete, vague, or difficult to interpret. Fragrance-related compounds were often grouped under generic terms such as “fragrance” or “parfum,” preventing individuals from identifying specific exposure risks. This lack of transparency may have contributed to accidental exposures and undermined participants’ ability to navigate indoor environments safely. Even products marketed toward sensitive populations frequently lacked sufficient disclosure regarding chemical content or airborne emissions.

- Sub-theme 2: Mislabelling and greenwashing

Participants also described widespread mislabelling and greenwashing, particularly involving “products marketed as ‘natural,’ ‘eco-friendly,’ ‘green,’ or ‘unscented.’” These labels were often perceived as misleading because products still contained fragrance-related chemicals or other irritants despite appearing safer. This inconsistency may have reinforced distrust of both manufacturers and institutional policies relying on self-regulated product claims.

- Sub-theme 3: Terminology

Participants reported that inconsistencies in policy terminology were associated with misunderstandings regarding which products or behaviors were actually prohibited. They further described this confusion surrounding policy and product terminology as a factor that may have weakened implementation. Participants also described uncertainty regarding the meaning of terms such as “scent-free,” “fragrance-free,” and “unscented,” noting that these labels were used inconsistently across organizations and products. In practice, unclear terminology contributed to misunderstandings about compliance requirements and allowed continued use of products perceived as incompatible with fragrance-free environments. These ambiguities may have reinforced earlier failures in education and enforcement by limiting shared understanding of what constituted a safe or accessible environment.

- Sub-theme 4: Commercial practices

Our findings revealed broader commercial practices, including aggressive fragrance marketing and normalization of scented products in public environments, that reinforced these exposures. Fragranced products were widely embedded within cleaning products, cosmetics, detergents, air fresheners, and retail environments, making exposure difficult to avoid in everyday settings. This normalization may have contributed to resistance toward fragrance-free practices and weakened institutional willingness to enforce fragrance-free policies.

- Sub-theme 5: Scent-free signs

Scent-free signs were found as frequently ineffective due to inconsistent placement, vague wording, or lack of enforcement. Although signs were common in some workplaces and healthcare settings, they were often ignored or treated as optional recommendations rather than accessibility measures. In many cases, heavily fragranced environments persisted despite visible scent-free messaging. Participants therefore viewed signage alone as insufficient without accompanying education, environmental controls, and accountability mechanisms to support meaningful compliance.

#### 3.3.5. Financial Barriers

Financial barriers emerged as a structural consequence of environmental inaccessibility, reflecting the economic impacts of institutional fragrance exposure. Respondents described the financial burden of avoiding exposures as largely individualized, requiring them to personally absorb the costs associated with creating safer indoor environments, accessing healthcare, and maintaining daily functioning. These economic pressures frequently intersected with reduced employment capacity, housing instability, and limited access to disability-related support.

- Sub-theme 1: Costly safe and alternative materials/products

One of the most commonly reported issues involved the high cost of safe and alternative materials or products. Substantial expenses were associated with purchasing fragrance-free cleaning products, lowest-emission household items, air filtration systems, specialized personal protective tools, and alternative housing arrangements. Because safer products were often less available and more expensive, participants frequently faced difficult trade-offs between health protection and financial stability. These costs were compounded by the need to continually avoid exposure in environments where fragranced products were still widely used.

- Sub-theme 2: Low to no financial support

Participants described low to no financial support for accommodations, environmental modifications, healthcare expenses, or disability-related needs. Many reported limited access to workplace accommodations, housing support, or reimbursement for exposure-reduction measures, despite experiencing significant functional impairment. Financial pressures were further intensified by reduced work participation, job instability, and loss of income associated with inaccessible indoor environments and repeated exposure-related illness. These findings illustrate how economic barriers reinforced earlier implementation failures. Weak enforcement, poor environmental controls, and limited availability of safer alternatives shifted responsibility for preventing exposure onto individuals, requiring participants to bear the costs of accessibility. As a result, financial vulnerability became both an outcome of systemic policy failure and an additional barrier limiting participants’ ability to protect themselves from harmful indoor environments.

### 3.4. Themes in Impacts: Health, Social, and Everyday Consequences

A broad range of health impacts was found to be associated with exposure to fragranced products, indoor pollutants, and poorly controlled indoor environments. These impacts were often multisystemic, recurrent, and closely connected to the accessibility barriers identified in previous sections.

#### 3.4.1. Physical Impacts

The most frequently discussed physical impacts were neurological symptoms, including migraines, headaches, numbness, and hearing disturbances. Cognitive symptoms, including brain fog, dizziness, confusion, and disorientation, were commonly reported following exposure to fragranced products and indoor pollutants and often interfered with participants’ ability to work, communicate, and complete routine tasks. Participants also described gastrointestinal symptoms such as nausea, vomiting, and stomach pain, frequently occurring alongside headaches and dizziness. Respiratory symptoms included coughing, sore throat, asthma exacerbations, breathing difficulties, and anaphylactic reactions. Some reported living with multiple overlapping conditions, including asthma, fibromyalgia, myalgic encephalomyelitis/chronic fatigue syndrome, migraines, dermatitis, and mast cell activation syndrome, emphasizing that symptoms were cumulative and interconnected rather than isolated within a single bodily system. Symptoms could persist for hours or days after exposure, contributing to chronic exhaustion, social withdrawal, and ongoing difficulties navigating everyday indoor environments.

#### 3.4.2. Psychological Impacts

Significant psychological impacts were linked to the ongoing exposure, inaccessible indoor environments, and repeated failures in accommodation and policy implementation. There was persistent anticipatory fear and anxiety related to entering indoor environments where exposure could occur. Many linked depressive symptoms to the loss of employment, restricted mobility, diminished independence, and inability to safely participate in previously routine activities. Ongoing barriers to healthcare access and repeated failures in accommodation may have further reinforced feelings of discouragement and emotional exhaustion. These experiences were intensified by persistent exposure within environments that formally claimed to be fragrance-free or accessible, creating a disconnect between policy expectations and lived experience. Participants also described feeling invalidated and socially excluded, often being perceived as difficult or unreasonable because of their accommodation needs and avoidance behaviors. One participant explained that it felt “somewhat embarrassing to have to ask” for accommodations, questioning why they needed to be treated as “special.” Another participant explained, “Some days it drags me down so much that I think, I can’t do this anymore”, illustrating the cumulative emotional burden associated with persistent exposure and limited escape from triggering environments.

#### 3.4.3. Social Impacts

Social isolation emerged as one of the most pervasive impacts described by participants. Their social lives had become extremely restricted, often limited to phone calls, online interactions, or a very small number of supportive individuals. Some participants reported losing friendships or romantic relationships and becoming increasingly dependent on partners, family members, or close support networks to complete everyday activities and navigate potentially unsafe environments. One participant summarized this experience by stating that “when you’re disabled, you’re needy, you need people”, highlighting the emotional complexity associated with increased dependence. Another participant described working in an isolated office space that improved their physical health but significantly harmed their social well-being, suggesting exposure avoidance strategies often came at the cost of meaningful social engagement and community participation. They also explained that leaving a relatively safe home after more than twenty years would be “really quite devastating” because accessible housing options were extremely limited. These findings highlight how failures in IAQ regulation, housing protections, and environmental control can threaten residential stability and contribute to ongoing insecurity for individuals with MCS and related conditions.

#### 3.4.4. Financial Impacts

Loss of income emerged as one of the most significant financial consequences associated with chronic exposure and inaccessible indoor environments. Worsening symptoms, cognitive difficulties, and repeated exposure in workplaces may have ultimately made continued employment impossible. One participant explained that workplace exposures became so severe that they “ended up having to quit work,” while another described making errors at work before realizing that their symptoms were linked to environmental exposures. The financial consequences of employment loss often extended into housing instability and reduced ability to maintain safer indoor environments. Some individuals further described the “double-edged sword” in which illness prevented continued employment while reduced income simultaneously limited their ability to afford healthier housing or environmental modifications. Also, they reported selling homes, moving into less suitable housing, or becoming increasingly financially vulnerable after leaving work due to MCS-related illness.

### 3.5. Themes in Solutions: Pathways Toward Inclusive and Effective Fragrance-Free Policy Implementation

Respondents proposed a range of solutions aimed at reducing exposure, improving accessibility, and strengthening institutional responses to IAQ-related disabilities. These solutions directly reflected the barriers and systemic failures identified throughout the study, particularly gaps in environmental control, policy enforcement, healthcare accessibility, and public awareness. Rather than focusing solely on individual behavior change, they emphasized the need for structural and environmental interventions capable of creating safer and more accessible indoor environments.

#### 3.5.1. Accessibility and Accommodation

Accessibility for individuals with MCS and related conditions required the ability to safely participate in indoor environments without harmful exposure. Environmental control emerged as one of the most important accommodations identified by participants, as they consistently emphasized the importance of being able to manage air quality, limit fragrance exposure, and regulate shared spaces and ventilation systems in both home and workplace environments. One participant noted that “having access to true scent-free products would be a bonus,” but stressed that making them affordable was equally important because “money is an issue” for many families. Positive experiences were associated with individualized accommodations that improved participation within educational, workplace, and healthcare settings. Remote work was highlighted as dramatically improving health and safety; as one participant explained, “I work from home now, where I’ve been safe ever since.” Overall, many of the proposed accommodations directly addressed the systemic failures identified earlier in the study, particularly inadequate environmental protections, weak enforcement, and limited recognition of IAQ-related disabilities.

#### 3.5.2. Awareness and Education

Educational initiatives were viewed as necessary to reduce stigma, improve accommodation practices, and strengthen compliance with fragrance-free policies. Increased awareness was viewed as essential for improving understanding of the legitimacy of IAQ-related disabilities and reducing skepticism surrounding accommodation requests. Participants specifically called for education targeting employers, healthcare providers, policymakers, educators, and the general public.

#### 3.5.3. Environmental Design Modifications

Multiple environmental and building-related interventions aimed at reducing exposure within indoor spaces were proposed, emphasizing the need for controlled indoor environments where exposure sources could be minimized and environmental conditions more consistently monitored. Central to these strategies was the replacement of fragranced and other VOC-emitting consumer products with fragrance-free, verified low-emission, or least-toxic alternatives, together with the selection of lowest-VOC-emitting paints, flooring, furniture, adhesives, and renovation materials to minimize off-gassing during construction and maintenance. Reduced occupancy and less crowded settings were also seen as beneficial for minimizing airborne exposure and improving environmental control. In addition, participants highlighted the importance of cleaner outdoor environments as outdoor pollutants and combustion-related emissions can enter indoor spaces and contribute to cumulative exposure. Structural interventions such as separate HVAC systems, reduced air recirculation, and properly designed ventilation systems with high-quality air filtration were consistently identified as critical measures to reduce airborne contaminants and improve IAQ in workplaces, housing, healthcare facilities, and public buildings.

#### 3.5.4. Healthcare Solutions

Participants’ accounts highlighted substantial gaps in healthcare accessibility, with numerous recommendations focused on reducing exposure and increasing institutional recognition of MCS and IAQ-related disabilities. These recommendations directly reflected earlier findings regarding healthcare inaccessibility, lack of provider awareness, inconsistent accommodation practices, and inadequate environmental protections. Participants emphasized the need for healthcare providers and clinics specializing in MCS and environmentally linked illnesses, while expressing frustration that healthcare environments themselves are often heavily fragranced or poorly controlled. As one participant noted, healthcare and educational settings should be required to use “the safest fragrance-free option,” highlighting the role of these institutions in protecting vulnerable populations and modeling public health leadership. Increased medical education, professional training, and research were also identified as critical for improving diagnosis, treatment, and clinical recognition, with one participant stating, “we need to be educating the doctors.” Stronger, consistently enforced fragrance-free policies and the mandatory use of the least-toxic products in healthcare settings were viewed as essential accessibility measures. Finally, formal recognition of MCS within healthcare and institutional frameworks was repeatedly emphasized as necessary to ensure access to accommodations, services, and legal protections. Although MCS is recognized within human rights and accessibility frameworks, participants described persistent challenges in obtaining consistent recognition and accommodation in practice.

#### 3.5.5. Personal Protective Measures

A common coping strategy involved substantial lifestyle modifications to minimize exposure to fragranced products, combustion-related pollutants, and poorly ventilated indoor environments. Many altered shopping habits, avoided public spaces, changed household products, modified clothing choices, or restricted social activities to reduce symptoms. Home environments were also extensively modified. Several participants described using a wide range of protective tools to reduce exposure and manage symptoms. One participant stated, “I always wear a mask, whether we need to or not,” while another explained that they had worn masks even before the COVID-19 pandemic.

#### 3.5.6. Policies and Regulations

Participants emphasized that meaningful improvements in accessibility and IAQ would require stronger policies, regulatory oversight, and enforceable environmental standards. They proposed measures such as zoning changes, alternative housing initiatives, and subsidies for fragrance-free products and safer materials, noting that these alternatives are often more expensive or difficult to obtain. Some expressed frustration that housing providers, including nonprofit housing organizations, were not always required to comply with accessibility-related environmental protections. The need for mandatory ingredient disclosure and clearer labeling standards was also repeatedly highlighted. The use of vague terms such as “fragrance” or “natural aroma” was criticized, arguing that these labels obscure important chemical information and limit informed decision-making. In addition, significant gaps in building standards, ventilation requirements, and IAQ regulations were identified. Several respondents reported being told that there were effectively “no standards for indoor air” to determine unsafe exposure levels, underscoring perceived regulatory deficiencies. As a result, they strongly supported enforceable fragrance-free policies across institutions but emphasized that existing policies often fail due to limited public understanding of what “fragrance-free” entails. Additionally, they stressed the importance of clear enforcement mechanisms and consequences for non-compliance. As one participant stated, “There needs to be something that’s going to stop people from doing it,” while another argued that fragrance-free policies should be enforced similarly to smoke-free regulations because “the health effects are the same.” Others proposed formal measures such as mandatory agreements during hiring processes and disciplinary actions for repeated non-compliance in workplace settings.

## 4. Discussion

This study examined lived experiences of IAQ accessibility barriers and participants’ perspectives on the implementation of fragrance-free policies and related IAQ practices. Our findings suggest that although fragrance-free policies are implemented in many workplaces, healthcare settings, and public environments, their implementation frequently fails to achieve consistent exposure reduction. These findings are consistent with a recent scoping review of fragrance-free policies, which found substantial variation in policy implementation and effectiveness, with many policies relying primarily on voluntary compliance rather than structured environmental control measures [8], suggesting that policy adoption alone may be insufficient to reduce fragrance-related exposures without clear implementation procedures and accountability measures.

Ongoing exposure to fragranced products, inadequate environmental controls, weak enforcement, and continued barriers to participation suggest that many existing fragrance-free policies function primarily as symbolic rather than operational measures. Collectively, these findings suggest that the presence of a fragrance-free policy does not necessarily translate into an accessible indoor environment. Accessibility was influenced not only by the existence of a policy, but by how consistently environmental controls, procurement practices, education, monitoring, and enforcement mechanisms were implemented. Many environments labeled as “fragrance-free” remained heavily fragranced due to scented laundry products, personal care products, cleaning agents, building materials, and inadequate ventilation. Structural measures were identified—including procurement controls, mandatory fragrance-free product standards, ventilation and air filtration improvements, staff education, environmental monitoring, and enforceable accountability measures—as necessary for meaningful accessibility and exposure reduction. These findings suggest that fragrance-free policies operate along a spectrum from symbolic recognition to functional environmental control. Symbolic policies rely primarily on signage and voluntary compliance, whereas functional policies incorporate procurement controls, enforcement mechanisms, environmental management practices, and structured education [8]. The symbolic-functional distinction identified in this study aligns closely with the IAQ hierarchy of controls, which emphasizes that source control and environmental interventions are more effective than strategies relying primarily on individual behavior change [12,13].

Taken together, the findings suggest that implementation failure occurs through interconnected pathways involving policy ambiguity, inadequate education, weak enforcement, limited environmental controls, and insufficient institutional accountability. These factors interact to create persistent exposure despite the formal presence of fragrance-free policies. Rather than representing isolated deficiencies, participants described a cascading pattern in which weaknesses in policy design and implementation reinforced one another, ultimately reducing accessibility and limiting the effectiveness of existing environmental protections.

Our findings further suggest that fragrance-related accessibility barriers extend beyond perfume use alone and are deeply embedded within broader indoor environmental systems. Converging IAQ evidence strengthened interpretation of these findings by demonstrating that fragranced products and indoor pollutant sources contribute to measurable VOC emissions and airborne contaminant exposure within indoor environments [1,12,14,15]. The convergence between lived experience and environmental evidence indicates that current policy approaches remain insufficient to consistently reduce exposure in shared indoor environments. These findings support the recognition of environmental exposures as accessibility barriers and reinforce the need to address IAQ within broader accessibility, inclusion, and human rights frameworks.

The consequences of inaccessible indoor environments extended beyond physical symptoms due to reduced employment opportunities, barriers to healthcare access, social isolation, financial hardship, and limitations in community participation. These outcomes are consistent with contemporary accessibility frameworks, which recognize that disability arises not solely from individual health conditions but from barriers within social and physical environments that restrict equitable participation. The findings therefore suggest that improving IAQ is not only an environmental health objective but also an accessibility and inclusion strategy capable of supporting participation across multiple domains of daily life.

We found that policies focused narrowly on perfume while overlooking cleaning and laundry products, air fresheners, and fragranced building materials. This finding aligns with broader IAQ research demonstrating that exposure sources extend beyond personal fragrances and include cleaning products, laundry products, air fresheners, building materials, renovation activities, and other indoor pollutant sources. This ambiguity, arising from narrow policy focus, contributed to uncertainty regarding policy expectations and educational failures, where individuals were instructed not to wear “fragrance” without understanding which products or exposures were relevant. Poor education then contributed to non-compliance, weak enforcement, and normalization of ongoing exposure within supposedly fragrance-free environments. This cascading implementation failure indicates that fragrance-free policies cannot function effectively when foundational policy components remain unclear or inconsistently applied.

Although this study focused primarily on fragrance-free policies, the findings suggest that effective environmental accessibility requires broader source-control approaches to IAQ. In addition to fragranced products, the findings suggest that broader indoor air quality and accessibility policies should also address other preventable indoor environmental exposures—including tobacco and cannabis smoke, pesticides and pest control products, cleaning and disinfecting agents, renovation-related emissions, mold and moisture-related contaminants, and other avoidable airborne pollutants—that may compromise accessibility for susceptible individuals. Fragrance-free policies should therefore be viewed as one component of a comprehensive indoor air quality and accessibility framework rather than as a standalone intervention.

Recent amendments to Health Canada’s cosmetic labeling requirements requiring disclosure of specified fragrance allergens represent an important step toward improving product transparency [16]. However, these requirements remain limited to designated fragrance allergens and do not address the broader range of VOC-emitting compounds, sensitizers, or other chemicals that may contribute to indoor air pollution and symptom triggering among susceptible individuals. The present findings, together with the related IAQ assessment study, suggest that effective fragrance-free procurement requires product selection based on verified low-emission characteristics rather than fragrance allergen disclosure alone.

Participants consistently described exclusion from workplaces, healthcare settings, educational institutions, public transportation, housing, and social environments because indoor spaces remained inaccessible despite the existence of formal policies. Consistent with accessibility legislation and disability frameworks, accessible environments require conditions that permit meaningful participation without disproportionate harm or exclusion [17,18]. Evidence demonstrates that environments containing persistent airborne triggers can function as disabling environments in ways comparable to other inaccessible physical spaces. These findings are consistent with the United Nations Committee on the Rights of Persons with Disabilities’ recognition of multiple chemical sensitivity within its 2025 Concluding Observations on Canada under Article 5 (Equality and Non-Discrimination), highlighting the need to address barriers that limit equal participation and accessibility for individuals living with MCS [19].

Moreover, participants did not frame accessibility as a matter of personal preference or lifestyle choice. Rather, exposure avoidance was repeatedly described as necessary for maintaining basic functioning, healthcare access, employment, and social participation. These findings support increasing recognition within environmental health and disability literature that MCS extends beyond individual symptom experiences and functions as an accessibility issue shaped by environmental conditions, institutional practices, and barriers to participation [2,20].

Our study findings additionally reveal important policy implications regarding environmental governance and consumer product regulation. Participants consistently identified gaps in ingredient disclosure, chemical testing, product labeling, VOC regulation, and building standards, and proposed precautionary regulatory approaches, mandatory disclosure systems, lowest-emission procurement standards, and strengthened IAQ-focused building regulations, reflecting broader environmental health literature emphasizing the importance of upstream exposure reduction rather than downstream management of individual symptoms [1,13,21].

Ultimately, the findings indicate that fragrance-free policies cannot function effectively when they rely primarily on symbolic gestures, voluntary compliance, or individual accommodation requests without corresponding structural controls [8]. Functional implementation requires integrated environmental management approaches that include procurement controls, ventilation standards, product regulation, education, monitoring, and enforceable accountability mechanisms [22]. More broadly, fragrance-free policies should be understood not as optional wellness initiatives, but as accessibility measures embedded within larger IAQ and disability frameworks.

The study relied on self-reported experiences and perceptions, which may reflect individual variability in symptom presentation and exposure interpretation. Given the cross-sectional study design, causal relationships between fragrance-free policies and reported health or accessibility outcomes cannot be established. Reverse causation is also possible, as workplaces with a higher prevalence of employees with MCS or related health concerns may have been more likely to adopt fragrance-free policies. As individuals with MCS or fragrance-related health concerns may have been more likely to work in, remain employed in, or participate in the survey from workplaces with fragrance-free policies, selection effects cannot be excluded. Accordingly, comparisons between respondents from workplaces with and without fragrance-free policies in this study should be interpreted as descriptive rather than as evidence of the health effects or effectiveness of these policies. However, the purpose of the study was to examine how participants experience and interpret accessibility barriers within indoor environments, rather than establishing direct causal relationships between specific exposures and health outcomes. Despite all the limitations, the study provides important evidence regarding the gap between fragrance-free policy intent and lived implementation. Across multiple environments, participants described continued exposure, ongoing exclusion, and the need to adopt extensive personal protective strategies despite the existence of formal policies. The convergence of qualitative findings, policy perceptions, and IAQ evidence suggests that current approaches remain insufficient to produce consistently accessible indoor environments.

## 5. Conclusions

Our findings suggest that fragrance-free policies should be viewed as structural accessibility interventions within broader environmental health and human rights frameworks. The convergence of lived-experience findings further demonstrates that current approaches frequently leave individuals with MCS and related conditions exposed, excluded, and unable to fully participate in workplaces, healthcare settings, public environments, and social life. Exploratory analyses indicate that the survey did not provide evidence of measurable differences associated with policy status within this sample. Moving forward, meaningful progress will require shifting from symbolic recognition toward structural implementation grounded in environmental health evidence, accessibility principles, and institutional accountability. Fragrance-free policies must be integrated within broader IAQ frameworks that prioritize source control, safer procurement practices, ventilation standards, environmental monitoring, public education, and enforceable implementation mechanisms. Without these structural interventions, individuals with MCS and related conditions will likely continue to experience preventable exposure, exclusion, and barriers to full participation in workplaces, healthcare settings, public environments, and daily life. Improving IAQ through source-control strategies represents one of the most practical and scalable approaches to advancing accessibility for individuals affected by fragrance and chemical exposures.

## Data Availability

The original contributions presented in this study are included in the article/Supplementary Material. Further inquiries can be directed to the corresponding author.

## Supplementary Materials

The following supporting information can be downloaded at: https://www.mdpi.com/article/10.3390/ijerph23081077/s1, Table S1: Descriptive characteristics of study participants (N = 60); Table S2: Sociodemographic and workplace characteristics (N = 48); Table S3: Fragrance-free policy awareness, training, and compliance (fragrance-free buildings only, N = 32); Table S4: Indoor air quality perceptions and comfort (N = 48); Table S5: Self-reported symptoms and chronic health conditions among building occupant survey respondents, by fragrance-free policy status.

## Abbreviations

The following abbreviations are used in this manuscript:

IAQ	Indoor Air Quality
MCS	Multiple Chemical Sensitivity
VOC	Volatile Organic Compound
CCHS	Canadian Community Health Survey
