# Supplementary material for: Fragrance-Free Policies in Practice: Identifying Perceived Implementation Gaps Through Lived Experience in a Qualitative-Dominant Mixed-Methods Study
Source: Int J Environ Res Public Health. 2026 Aug 19;23(8):1077. doi: 10.3390/ijerph23081077 (PMC13513598; doi:10.3390/ijerph23081077)
Supplement: Supplementary file 1 [file ijerph-23-01077-s001.zip › FFP_1_Table S1.pdf]

Table S1. Descriptive characteristics of study participants (N = 60)

Characteristic	n (%)
Gender	
Women	54 (90%)
Men	4 (7%)
Diverse	2 (3%)
Age (in years)	
19 - 35	2 (3%)
36 - 50	22 (37%)
51 - 64	20 (33%)
≥ 65	16 (27%)
Province/Territory	
Ontario	20 (33%)
Quebec	17 (28%)
British Columbia	7 (12%)
Alberta	6 (10%)
Saskatchewan	2 (3%)
Nova Scotia	4 (7%)
Newfoundland and Labrador	1 (2%)
Yukon	1 (2%)
Race/Ethnicity	
White – North American	38 (63%)
White European	15 (25%)
Mixed heritage	4 (7%)
Others	2 (3%)
Prefer not to answer	1 (2%)
Employment status	
Employed	21 (35%)
Unemployed	37 (62%)
Prefer not to answer	2 (3%)
Household income (in CAD)	
< $10,000	5 (8%)
$10,000 – $14,999	4 (7%)
$15,000 – $24,999	11 (18%)
$25,000 - $49,999	10 (17%)
$50,000 - $74,999	9 (15%)
≥$100,000	11 (18%)
Prefer not to answer	10 (17%)
Language	
English	47 (78%)
French	13 (22%)
