# Supplementary material for: Fragrance-Free Policies in Practice: Identifying Perceived Implementation Gaps Through Lived Experience in a Qualitative-Dominant Mixed-Methods Study
Source: Int J Environ Res Public Health. 2026 Aug 19;23(8):1077. doi: 10.3390/ijerph23081077 (PMC13513598; doi:10.3390/ijerph23081077)
Supplement: Supplementary file 1 [file ijerph-23-01077-s001.zip › FFP_1_Table S2.pdf]

Table S2. Sociodemographic and workplace characteristics (N = 48)

Variable	Category	n	%
Age group (years)	18–24	1	2.1
	25–34	8	16.7
	35–44	17	35.4
	45–54	13	27.1
	55–64	8	16.7
	65 or older	1	2.1
Gender	Female	35	72.9
	Male	12	25.0
	Gender diverse	1	2.1
Race/ethnicity	White – North American	39	81.2
	White – European	4	8.3
	Other/mixed [a]	5	10.4
Recent immigrant (≤5 years)	Yes	3	6.2
	No	45	93.8
Role/affiliation	Employee	44	91.7
	Business owner/operator	3	6.2
	Visitor	1	2.1
Workspace configuration	Enclosed private office	26	54.2
	Open space, no partitions	7	14.6
	Open space, tall partitions (≥6 ft)	5	10.4
	Open space, short partitions (<6 ft)	4	8.3
	Other	5	10.4
	Not reported	1	2.1
Building sample policy status (N = 17 buildings)			
Distinct offices represented [b]	Fragrance-free	10	-
	Non-fragrance-free	7	-
Number of occupants per policy status	Fragrance-free	32	66.7
	Non-fragrance-free	16	33.3

Note: [a] Includes Latin American, First Nations/White, Asian – South East, Métis, and do not know (n = 1 each).
[b] Reflects offices with ≥1 survey respondent, out of 32 offices sampled study-wide for IAQ testing (see companion IAQ testing paper).
