# Supplementary material for: Fragrance-Free Policies in Practice: Identifying Perceived Implementation Gaps Through Lived Experience in a Qualitative-Dominant Mixed-Methods Study
Source: Int J Environ Res Public Health. 2026 Aug 19;23(8):1077. doi: 10.3390/ijerph23081077 (PMC13513598; doi:10.3390/ijerph23081077)
Supplement: Supplementary file 1 [file ijerph-23-01077-s001.zip › FFP_1_Table S3.pdf]

Table S3. Fragrance-free policy awareness, training, and compliance (fragrance-free buildings only, N = 32)

Variable	Category	n	%
Aware of fragrance-free policy	Yes	23	71.9
	No / not indicated	9	28.1
Received training or educational material [a]	Yes	1	5.6
	No	16	88.9
	Not sure	1	5.6
	Not reported	5	-
Personally complies with policy [b]	Yes	20	90.9
	No	2	9.1
	Not reported	1	-
Perceived compliance of other occupants [b]	High	9	42.9
	Moderate	9	42.9
	Low	3	14.3
	Not reported	2	-
Believes policy is properly monitored	Yes	9	42.9
	No	3	14.3
	Not sure	9	42.9
	Not reported	2	-
Believes non-compliance is addressed	Yes	6	31.6
	No	5	26.3
	Not sure	8	42.1
	Not reported	4	-
Supportive of fragrance-free policy	Yes	26	86.7
	Neutral / not sure	3	10.0
	No	1	3.3
	Not reported	2	-
Concerned about environmental impact of scented products	Yes	20	66.7
	No	10	33.3
	Not reported	2	-

Note: [a] Asked only of respondents who indicated awareness of the policy (n = 23).

[b] Perceived compliance recoded numerically (High = 3, Moderate = 2, Low = 1) among respondents who answered.
