# Supplementary material for: Fragrance-Free Policies in Practice: Identifying Perceived Implementation Gaps Through Lived Experience in a Qualitative-Dominant Mixed-Methods Study
Source: Int J Environ Res Public Health. 2026 Aug 19;23(8):1077. doi: 10.3390/ijerph23081077 (PMC13513598; doi:10.3390/ijerph23081077)
Supplement: Supplementary file 1 [file ijerph-23-01077-s001.zip › FFP_1_Table S4.pdf]

Table S4. Indoor air quality perceptions and comfort (N = 48)

Variable	Category	n	%
General IAQ perception	Excellent	15	33.3
	Good	27	60.0
	Fair	2	4.4
	Poor	1	2.2
	Not reported	3	—
Bothersome odors present in workspace	Yes	5	10.4
	No	43	89.6
Main source of discomfort [a] (of 43 mentions)	Temperature	25	58.1
	Humidity	8	18.6
	Poor air circulation	5	11.6
	Odors	3	7.0
	Dust	2	4.7

[a] Multiple selections permitted; percentages calculated over total discomfort mentions (not total respondents).
