# Supplementary material for: Fragrance-Free Policies in Practice: Identifying Perceived Implementation Gaps Through Lived Experience in a Qualitative-Dominant Mixed-Methods Study
Source: Int J Environ Res Public Health. 2026 Aug 19;23(8):1077. doi: 10.3390/ijerph23081077 (PMC13513598; doi:10.3390/ijerph23081077)
Supplement: Supplementary file 1 [file ijerph-23-01077-s001.zip › FFP_1_Table S5.pdf]

Table S5. Self-reported symptoms and chronic health conditions among building occupant survey respondents, by fragrance-free policy status

Variable	Fragrance-free n (%) N=32	Non-fragrance-free n (%) N=16	Combined n (%) N=48
**Self-reported symptoms in the past 4 weeks [a]**			
Migraine/headaches	17 (53.1)	2 (12.5)	19 (39.6)
Gastrointestinal problems	10 (31.2)	2 (12.5)	12 (25.0)
Cognitive problems	9 (28.1)	1 (6.2)	10 (20.8)
Respiratory problems	7 (21.9)	1 (6.2)	8 (16.7)
Musculoskeletal problems	5 (15.6)	2 (12.5)	7 (14.6)
Ocular problems	5 (15.6)	0 (0.0)	5 (10.4)
Skin problems	5 (15.6)	0 (0.0)	5 (10.4)
Immune system problems	4 (12.5)	0 (0.0)	4 (8.3)
Auditory problems	3 (9.4)	0 (0.0)	3 (6.2)
Cardiovascular problems	2 (6.2)	1 (6.2)	3 (6.2)
Asthma attacks	1 (3.1)	0 (0.0)	1 (2.1)
Neurological problems	1 (3.1)	0 (0.0)	1 (2.1)
**Chronic health conditions, ever diagnosed or self-diagnosed [b]**			
Eczema/skin problems	12 (37.5)	4 (25.0)	16 (33.3)
Allergy to dust/molds	13 (40.6)	1 (6.2)	14 (29.2)
Food allergy	12 (37.5)	2 (12.5)	14 (29.2)
Migraine	12 (37.5)	2 (12.5)	14 (29.2)
Asthma	9 (28.1)	0 (0.0)	9 (18.8)
Bowel disorders	8 (25.0)	1 (6.2)	9 (18.8)
Back problems	8 (25.0)	0 (0.0)	8 (16.7)
Hay fever	7 (21.9)	1 (6.2)	8 (16.7)
Thyroid disease	6 (18.8)	2 (12.5)	8 (16.7)
Multiple Chemical Sensitivity	6 (18.8)	1 (6.2)	7 (14.6)
Bronchitis	5 (15.6)	0 (0.0)	5 (10.4)
Arthritis/rheumatism	4 (12.5)	0 (0.0)	4 (8.3)
High blood pressure	3 (9.4)	1 (6.2)	4 (8.3)
Cancer	2 (6.2)	1 (6.2)	3 (6.2)
Autism/Asperger's	1 (3.1)	0 (0.0)	1 (2.1)
Cardiac problems	1 (3.1)	0 (0.0)	1 (2.1)
Chronic fatigue	1 (3.1)	0 (0.0)	1 (2.1)
Chronic obstructive pulmonary disease	1 (3.1)	0 (0.0)	1 (2.1)
Fibromyalgia	1 (3.1)	0 (0.0)	1 (2.1)
No known conditions	9 (28.1)	10 (62.5)	19 (39.6)

[a] Frequency response options collapsed to "reporting" (any frequency other than 'not in the last four weeks') vs. 'not reporting'; respondents who left an item blank were excluded from that item's denominator.

[b] Conditions collected via multi-select checklist; unselected items treated as absent.
